# The mediating effects of personality traits on the relationship of youth conduct problems and mood disorders with adulthood suicidality

**DOI:** 10.1038/s41598-023-31338-9

**Published:** 2023-03-15

**Authors:** Tsung-Yang Wang, Hsi-Chung Chen, Cheng-Dien Hsu, I-Ming Chen, Shih-Cheng Liao, Chiao-Erh Chang, Ying-Yeh Chen, Jen-Hui Chan, Po-Hsiu Kuo

**Affiliations:** 1 Department of Psychiatry, National Taiwan University Hospital, Taipei, Taiwan; 2 Department of Psychiatry, Taiwan Adventist Hospital, Taipei, Taiwan; 3 Institute of Epidemiology and Preventive Medicine, College of Public Health, National Taiwan University, Room 501, No. 17, Xu-Zhou Road, Taipei, 100 Taiwan; 4 Department of Psychiatry, Taipei City Psychiatric Center, Taipei City Hospital, Taipei, Taiwan; 5 Department of Psychiatry, National Yang-Ming University, Taipei, Taiwan; 6 National Taiwan University Hospital Hsin-Chu Branch, Hsinchu, Taiwan; 7 Psychiatric Research Center, Wan Fang Hospital, Taipei Medical University, Taipei, Taiwan

**Keywords:** Disease prevention, Public health, Epidemiology, Human behaviour, Social behaviour

## Abstract

Identifying the relevant factors for suicidality in individuals with conduct problems is a public health concern, especially if they were under the influence of mood disorders later in life. This study investigates the relationship between youth conduct problems and mood disorders and adulthood suicidality, and to further explore the mediating effects of personality on this relationship. A retrospective cohort study was administered to 308 individuals aged 20–65 years, with or without mood disorders diagnosed by psychiatrists. The Composite International Diagnosis Interview was used to evaluate conduct problems in youth and suicidality (i.e., suicide plan and suicide attempt) in the past year. Personality traits were assessed using Eysenck Personality Questionnaire-Revised for extraversion and neuroticism. Multiple-mediator analysis was used to investigate the mediation effects of personality traits on the relationship between conduct problems and suicidality. The average age of enrolled participants was 31.6 years, and 42.5% of them were female. 39.2% reported suicidality and 43.2% reported conduct problems in youth. Participants who were diagnosed with mood disorders (*p* < 0.001) and reported having conduct problems (*p* = 0.004) were associated with high suicidality. Multiple-mediator analysis showed that conduct problems in youth increased the risk of adulthood suicidality through the indirect effects of higher neuroticism (suicide plan: OR = 1.30, BCA 95% CI = 1.04–1.83; suicide attempt: OR = 1.27, BCA 95% CI = 1.05–1.66). Neuroticism mediates the association between youth conduct problems and adulthood suicidality. This finding raises our attention to assess personality traits in individuals with youth conduct problems for designing proper intervention strategies to reduce the risk of suicide.

## Introduction

Conduct disorder (CD) is a child and adolescent psychiatric disorder characterized by recurrent violent and destructive behaviors^1^. The prevalence of CD has been estimated to be 3–10% in school-aged children and may be as high as 60% in juvenile detention facilities^2^. Over 20 years of follow-up, early studies have demonstrated that a proportion of adolescents with conduct problems develop anxiety, substance abuse, psychosis, or even antisocial personality disorders^3^. Moreover, with the high prevalence of CD in juvenile detention facilities, the prevalence of current suicide ideation has been reported to be high in these facilities, ranging from 10.3 to 30%^4–7^.

Cohort and twin studies have demonstrated the potential link between CD and suicidality. The Finnish 1981 Birth Cohort Study, which followed up 5302 adolescents and collected national register-based information on suicide until the age of 24 years, reported that the occurrence of conduct problems at the age of 8 years increased the likelihood of suicide attempts and completed suicides in men^8^. Similar findings of increased suicidal thoughts and attempts were reported for sixth graders followed up to middle school^9^. Conduct problems dose dependently increased the risk of suicide attempts in female participants^10^. The link between CD and suicide was also noted in clinical settings. A previous study examined youths aged 12–19 years who were admitted to psychiatric units in the United States and showed that CD increased the risk of suicide attempts across 13 years of follow-up^11^. However, the mechanisms underlying this link were unclear. Twin studies have provided some clues suggesting that not only genetic factors contribute to the association between conduct problems and heightened suicidality^12,13^. Suicidality in individuals with conduct problems is a public health concern, and the relevant factors for suicidality in these individuals should be identified.

The related studies have mostly been conducted during adolescence and young adulthood, but suicidal behaviors can occur throughout midlife or even much later. The mechanisms underlying the link between conduct problems manifested at a younger age and suicide in adulthood remain unclear, especially in individuals who develop mood disorders later on; suicidal behaviors are a concern in these individuals. Past longitudinal studies showed that conduct problems and other psychiatric problems in childhood could persist into adulthood with negative impacts^14–16^. Childhood psychiatric problems could lead to adulthood adversity including suicidality even if the problems are subthreshold and best predicted by accumulative childhood exposure^16^. The high correlation of CD with mood disorders has been frequently reported^17^. However, no differences were found in the factors (i.e., the impulsivity of CD and the symptoms of mood disorders) contributing to the risk of suicidal behavior between CD and mood disorders. So far, the findings regarding the influence of mood disorders on the link between CD and suicidal behaviors have been inconclusive. Goldston et al.^11^ found that the influence of CD on the risk of suicide attempts was only prominent in individuals with depression, whereas another study did not find any synergistic effects of depression and conduct problems on suicide ideation or attempts^9^. The Finnish cohort study showed that self-reported depression did not predict suicidality among adolescents with conduct problems^8^. Some of these studies have only explored suicide thoughts as the outcome measures, and other studies have examined attempted or completed suicides as the outcome measures. So far, the mechanism underlying the roles of the comorbidity of CD and mood disorders in suicidality has not been well studied. Despite the aforementioned inconsistent findings, most studies have evidenced that conduct problems and mood disorders should be considered simultaneously in youth suicide prevention^11,12,18^.

Also, personality traits such as neuroticism, introversion, and cluster B or C traits were reported to be related to suicidality in different populations^19,20^. If the antisocial behaviors of children with conduct disorder persisted until adulthood, the diagnosis would be converted to antisocial personality disorder^21^. Antisocial personality traits, such as aggressiveness, anger, destructive behavior, impulsivity and sadistic traits, were found to be connected to suicidal behavior among adolescents^22^. Adulthood psychopathy is also a relevant factor for increased suicidality^23–25^. However, other dimensions of personality might play a role in connecting youth and adult mental problems. An Indian study reported higher psychoticism and neuroticism scores found in children with CD^26^. Aside from antisocial personality traits, whether other personality traits mediate the association between youth conduct problems and adulthood suicidality is unclear. We hypothesized that adults who have had conduct problems in adolescence or childhood have increased suicidality risk, and the effects were mediated via personality traits. In this study, we investigated the risk of suicide plans or even suicide attempts among adults with conduct problems in youth to evaluate the long-term risk in individuals with or without mood disorders. Moreover, through mediation analysis, we evaluated whether personality traits mediate the relationship between CD and suicidal outcomes.

## Methods

### Participants

We recruited patients and healthy participants aged 20–65 years with a mean age of 31.6 ± 7.7 years old. All participants received NT$200 for participation. Psychiatric outpatients from six general and psychiatric hospitals and healthy participants residing in the same community of the hospitals were invited to participate in this study from 2008 to 2010. Patients had been diagnosed with major depressive disorder (MDD) or bipolar affective disorder (BAD, including type I and type II) by psychiatrists according to Diagnostic and Statistical Manual of Mental Disorders, fourth edition, text revision (DSM-IV-TR) and were currently not experiencing acute episodes. Participants who had ever diagnosed with schizophrenia, organic psychosis, or substance-induced mood disorder or whose diagnosis changed during the study period were excluded. Healthy participants had no psychiatric history or related medical record and no intellectual disabilities. Eligible participants were interviewed by trained assistants by using the Chinese version of Composite International Diagnostic Interview (CIDI)^27^ to confirm their diagnoses of mood disorders and to collect information on demographic and clinical characteristics, including sex, age, marital status, educational level, occupation, alcohol use, smoking, conduct problems in youth, and substance misuse. To evaluate personality traits, all participants were asked to complete the Eysenck Personality Questionnaire-Revised Short Scale (EPQ-R Short Scale)^28^. The recruitment protocol was approved by the National Taiwan University Hospital Research Ethics Committee Office and Mackay Memorial Hospital Institutional Review Board. All research methods were performed in accordance with relevant guidelines and regulations. Informed consent was obtained from all participants prior to the study.

### Conduct problem and suicidality

The symptoms of CD in youth were retrospectively evaluated using 19 yes/no items in the Conduct Disorder section of CIDI^29^. The 19 items were corresponding to 15 criteria of CD in DSM-5; 4 of them had 2 pertinent items in the CIDI to double-check the answers. These four criteria included ‘Stolen while confronting a victim,’ ‘Lies to obtain goods or favors or to avoid obligations, ‘Stolen items of nontrivial value without confronting a victim,’ and ‘Run away from home overnight.’ The number of conduct problems was summed over the 15 criteria in DSM-5, which ranged from 0 to 15. A dichotomous variable of conduct problems was defined as three or more positive symptoms in this study as a broader representation of conduct problems in youth than just the diagnosis of conduct disorder. We also considered a more stringent definition with a cutoff of four or more symptoms based on DSM 5-TR, and performed a sensitivity analysis to evaluate the robustness of the main findings.

The three modules in the Suicide section of CIDI were used to assess the suicidality of all participants. The modules assessed if participants had suicide ideation, suicide plans, or suicide attempts at any time point in the past year. Among our participants, those who had suicide attempts or suicide plans all reported suicidal ideas (Fig. S1). The definition of suicidality used in this study was hierarchical, and suicidality was analyzed in the order of suicide attempts, suicide plans, and no suicide experiences. Participants who replied YES to “YOU ATTEMPTED SUICIDE” would be categorized as suicide attempts; those who replied YES to “YOU MADE A PLAN FOR COMMITTING SUICIDE” would be categorized as suicide plans; those who replied No to both questions would be categorized as no suicide experiences.

### Personality traits

We used the Chinese version of EPQ-R Short Scale to measure personality traits, which originally consisted of 36 items in the dimensions of neuroticism, extraversion, and psychoticism. We previously conducted psychometric analysis of the Chinese version of EPQ. The neuroticism and extraversion dimensions had high reliability and validity, but not the psychoticism dimension. Therefore, only the neuroticism and extraversion dimensions were applied in this study^28^. Participants with high extraversion are more sociable, dominant, and active, whereas those with high neuroticism exhibit more anxiety, guilt, low self-esteem, and lack of autonomy.

### Statistical analysis

Statistical analyses were performed using IBM SPSS version 20 (SPSS Inc., Chicago, IL, USA). There was no missing data in our dataset due to the rigorous recruitment process. The χ^2^ test or analysis of variance was used for univariate analysis to examine the suicidality and conduct problems by sociodemographic and clinical characteristics. A *p* value of < 0.05 was regarded as statistically significant. The variables that showed significant associations with both suicidality and conduct problems were used as covariates in the subsequent multinomial logistic regression and mediation model to adjust for confounding effects. The presence of conduct problems was defined as two models, including dichotomous and continuous measures to further validate whether the relationship between conduct problems and suicidality is linear. The mediating effects of personality traits (neuroticism and extraversion) on the relationship between conduct problems and suicidality were examined using a multiple-mediator model through an SPSS macro provided by Preacher and Haynes^30^, which controls for the confounding effects of covariates simultaneously. We used a bootstrapping strategy to test the validity of direct and indirect effects. In this study, indirect effects were bootstrapped with 5000 samples. These effects were bias-corrected, and a bias-corrected and accelerated 95% confidence interval (BCA 95% CI) was estimated. Current evidence suggests that for testing the indirect effect, bootstrapping methods are superior to methods that assume symmetry or normality of the sampling distribution^31^.

## Results

A total of 308 participants were enrolled in this study. Table 1 presents their sociodemographic and clinical profiles. The average age of participants was 31.6 ± 7.7 years, and 42.5% of them were female. Moreover, 55.8% of the participants were unemployed, and only 29.5% were married. Regarding psychiatric diagnosis, 11.4% of the participants were diagnosed with MDD, 53.6% were diagnosed with BAD, and 35.1% were healthy participants. Overall, 43.2% reported conduct problems in youth. The mean score of extraversion was 6.1 ± 2.6, and that of neuroticism was 7.0 ± 3.7. In the study population, 39.2% reported suicidality in the past year, including 9.7% with suicidal ideation and 29.5% with suicidal attempts. Univariate analysis revealed that the distribution of suicidality significantly differed between men and women (*p* < 0.001); men had more suicidal plans, and women had more suicidal attempts. Participants who were unemployed (*p* < 0.001), diagnosed with mood disorders (*p* < 0.001), reported having conduct problems (*p* = 0.004), and had high neuroticism (*p* < 0.001) were associated with high suicidality. Among participants who reported to have conduct problems, the mean number of symptoms was ranged from 2.3 with no suicide experience to 3.3 in participants with suicide attempts. The most commonly reported symptom was “lies to obtain goods or favors or to avoid obligations,” which was seen in 47.4% of individuals. In contrast, the symptom “forced someone into sexual activity” was rarely reported in only 1% of participants (Table S1).

**Table 1:** Sociodemographic characteristics of participants by suicide experience (n = 308).

	Total	No suicide experience	Suicide plans	Suicide attempts	*p*-value for Chi-square/ANOVA
		(n = 187)	(n = 30)	(n = 91)	
	n (%)	n (%)	n (%)	n (%)	
Age (years) (mean, SD)	31.6 (7.7)	32.0 (8.0)	28.7 (7.1)	31.9 (7.4)	0.09
Sex					
Female	131 (42.5)	69 (36.9)	8 (26.7)	54 (59.3)	< 0.001
Male	177 (57.5))	118 (63.1)	22 (73.3)	37 (40.7)	
Marital status					
Married	91 (29.5)	61 (32.6)	5 (16.7)	25 (27.5)	0.18
Separated/divorced/widowed/Single	217 (70.5)	126 (67.4)	25 (83.3)	66 (72.5)	
Education status					
Junior high school	59 (19.2)	28 (15.0)	2 (6.7)	29 (31.9)	0.001
Senior high school	169 (54.9)	103 (55.1)	17 (56.7)	49 (53.8)	
University	80 (26.0)	56 (29.9)	11 (36.7)	13 (14.3)	
Job					
Unemployed	172 (55.8)	85 (45.5)	21 (70.0)	66 (72.5)	< 0.001
Employed	136 (44.2)	102 (54.5)	9 (30.0)	25 (27.5)	
Alcohol drinking					
No drinking	189 (61.4)	123 (65.8)	16 (53.3)	50 (54.9)	0.26
Drinking without abuse	73 (23.7)	40 (21.4)	10 (33.3)	23 (25.3)	
Drinking with abuse	46 (14.9)	24 (12.8)	4 (13.3)	18 (19.8)	
Smoking status					
Non-smoker	122 (39.6)	81 (43.3)	10 (33.3)	31 (34.0)	0.052
Ex-smoker	86 (27.9)	57 (30.5)	9 (30.0)	20 (22.0)	
Current smoker	100 (32.5)	49 (26.2)	11 (36.7)	40 (44.0)	
Diagnosis					
Major depressive disorder + Bipolar disorder	200 (64.9)	94 (50.3)	23 (76.7)	83 (91.2)	< 0.001
No diagnosis	108 (35.1)	93 (49.7)	7 (23.3)	8 (8.8)	
Conduct problems					
Dichotomous (≥ 3 items)	133 (43.2)	76 (40.6)	13 (43.3)	44 (48.4)	0.48
Continuous (mean, SD)	2.7 (2.7)	2.3 (2.3)	3.2 (3.1)	3.3 (3.2)	0.004
Eysenck personality questionnaire (mean, SD)					
Extraversion	6.1 (2.6)	6.3 (2.6)	5.7 (2.7)	5.8 (2.5)	0.17
Neuroticism	7.0 (3.7)	5.6 (3.4)	8.5 (2.7)	9.3 (3.1)	< 0.001

### Conduct problems

Table 2 shows the correlations between conduct problems and sociodemographic and clinical profiles. Being male (*p* = 0.002), being separated/divorced/widowed/single (*p* = 0.02), being unemployed (*p* = 0.003), and having alcohol drinking (*p* = 0.001) and smoking habits (*p* < 0.001) were positively correlated with conduct problems in youth. Participants with conduct problems also reported significantly higher scores for extraversion (*p* = 0.01) and neuroticism (*p* = 0.002).

**Table 2:** Sociodemographic and clinical characteristics of participants by conduct problems (n = 308).

	Conduct problems (≥ 3 items)		*p*-value for Chi-square/ANOVA
	Without	With	
	n (%)	n (%)	
Age (years) (mean, SD)	31.9 (8.0)	31.3 (7.4)	0.49
Sex			
Female	88 (50.3)	43 (32.3)	0.002
Male	87 (49.7)	90 (67.7)	
Marital status			
Married	61 (34.9)	30 (22.6)	0.02
Separated/divorced/widowed/Single	114 (65.1)	103 (77.4)	
Education status			
Junior high school	30 (17.1)	29 (21.8)	0.54
Senior high school	100 (57.1)	69 (51.9)	
University	45 (25.7)	35 (26.3)	
Job			
Unemployed	85 (48.6)	87 (65.4)	0.003
Employed	90 (51.4)	46 (34.6)	
Alcohol drinking			
No drinking	123 (70.3)	66 (49.6)	0.001
Drinking without abuse	33 (18.9)	40 (30.1)	
Drinking with abuse	19 (10.9)	27 (20.3)	
Smoking status			
Non-smoker	86 (49.1)	36 (27.1)	< 0.001
Ex- smoker	50 28.6)	36 (27.1)	
Current smoker	39 (22.3)	61 (45.9)	
Diagnosis			
Major depressive disorder	20 (11.4)	15 (11.3)	0.16
Bipolar disorder	86 (49.1)	79 (59.4)	
No diagnosis	69 (39.4)	39 (29.3)	
Eysenck personality questionnaire (mean, SD)			
Extraversion	5.7 (2.6)	6.5 (2.5)	0.01
Neuroticism	6.4 (3.8)	7.7 (3.4)	0.002

### Conduct problems and suicidality

Table 3 shows the results of the multinomial logistic regression analysis of the relationship between conduct problems and suicidality. The presence of conduct problems was defined as two models. In model I, conduct problems were dichotomized by a cutoff of three in numbers. In model II, the total number of conduct problems was specified as a continuous variable. Conduct problems defined as a continuous variable was associated with a higher likelihood of suicide attempts (OR = 1.16; 95% CI = 1.06–1.27). After controlling for covariates, the association persisted (OR = 1.13; 95% CI = 1.01–1.27). Personality traits were correlated with suicidality. Neuroticism was associated with a higher risk of suicide plans both in model I (OR = 1.22; 95% CI = 1.06–1.41) and model II (OR = 1.21; 95% CI = 1.05–1.39). Also, higher neuroticism was associated with elevated risks of suicide attempts both in model 1 (OR = 1.28; 95% CI = 1.15–1.43) and model II (OR = 1.27; 95% CI = 1.14–1.41). In addition, female sex, unemployment, and diagnosis of mood disorders also significantly increased the odds for suicide attempts in both models I and II.

**Table 3:** Multinomial logistic regression analyses for factors associated with suicide-related experiences.

	Suicide plans			Suicide attempts		
		Model I	Model II		Model I	Model II
	Crude OR (95% CI)	Adjust OR (95% CI)	Adjust OR (95% CI)	Crude OR (95% CI)	Adjust OR (95% CI)	Adjust OR (95% CI)
Age (years)	0.95 (0.90–1.00)	0.98 (0.93–1.04)	0.98 (0.92–1.04)	1.00 (0.97–1.03)	1.05 (1.01–1.10)	1.05 (1.00–1.10)
Sex						
Female versus male	0.62 (0.26–1.47)	0.63 (0.25–1.61)	0.77 (0.30–1.96)	2.50 (1.49–4.17)	2.56 (1.34–4.89)	3.09 (1.60–6.00)
Job						
Unemployed versus employed	2.80 (1.22–6.44)	1.75 (0.69–4.41)	1.63 (0.65–4.09)	3.17 (1.84–5.45)	2.45 (1.25–4.78)	2.32 (1.19–4.54)
Conduct problems*						
Dichotomous (with versus without)	1.12 (0.51–2.43)	0.76 (0.32–1.80)	–	1.37 (0.83–2.26)	1.03 (0.53–1.99)	–
Continuous	1.14 (0.99–1.31)	–	1.09 (0.94–1.26)	1.16 (1.06–1.27)	–	1.13 (1.01–1.27)
Diagnosis						
Major depressive disorder versus no diagnosis	3.62 (0.82–16.08)	1.55 (0.31–7.75)	1.37 (0.27–6.96)	22.19 (7.95–61.95)	8.60 (2.76–26.80)	7.69 (2.45–24.18)
Bipolar disorder versus no diagnosis	3.20 (1.29–7.96)	1.58 (0.58–4.33)	1.47 (0.53–4.07)	8.68 (3.93–19.20)	5.13 (2.08–12.65)	4.78 (1.93–11.86)
Eysenck personality questionnaire						
Extraversion	0.91 (0.78–1.06)	0.96 (0.81–1.13)	0.92 (0.78–1.09)	0.92 (0.83–1.01)	1.01 (0.89–1.14)	0.98 (0.86–1.11)
Neuroticism	1.28 (1.13–1.45)	1.22 (1.06–1.41)	1.21 (1.05–1.39)	1.41 (1.28–1.55)	1.28 (1.15–1.43)	1.27 (1.14–1.41)

*Two definitions of presence of conduct problems were specified into the models separately. In model I, conduct problems were dichotomozed by ≥ 3 and < 3 in numbers, Nagelkerke's R^2^ = 0.325. In model II, the total numbers of conduct problems were specified into the model as a continuous variable, Nagelkerke's R^2^ = 0.334.

### Mediation analysis

**Figure 1:**
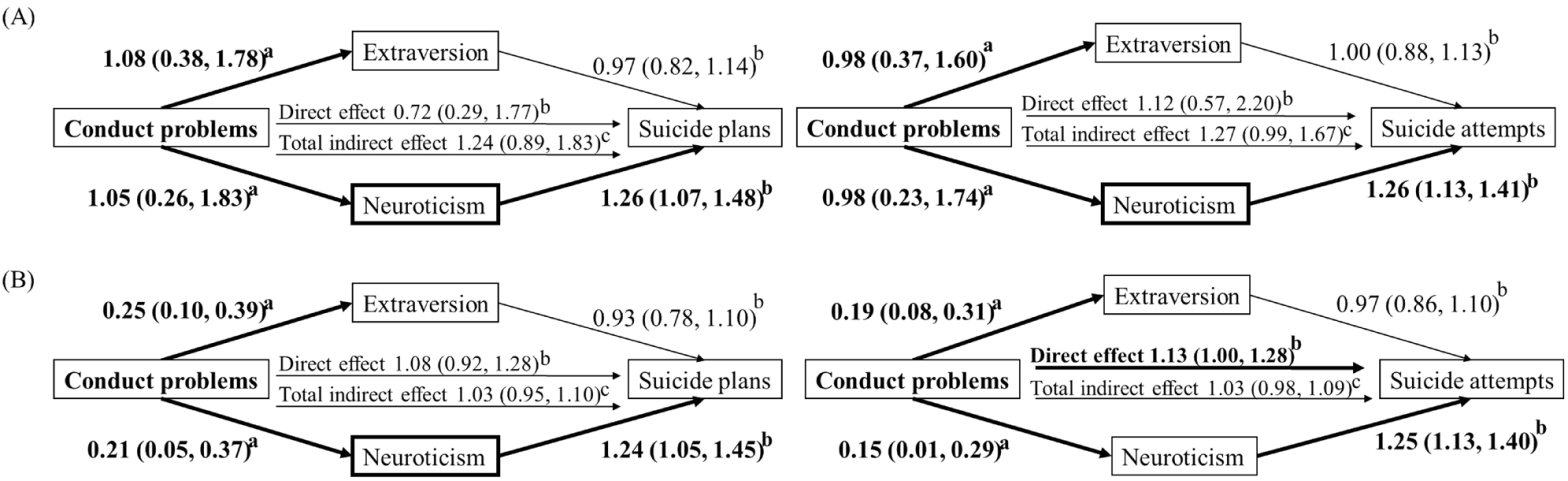
Pathways and corresponding coefficients of conduct problems that relate to suicidality. (**A**) Effects of dichotomous conduct problems on suicidality mediated by extraversion and neuroticism. (**B**) Effects of total numbers of conduct problems on suicidality mediated by extraversion and neuroticism. ^a^Coefficients and their 95% confidence intervals for linear regression analysis. ^b^Odds ratios and their 95% confidence intervals in the logistic regression analysis. ^c^Odds ratios and their bias-corrected and accelerated 95% confidence intervals in the logistic regression analysis. *Statistical significance < 0.05 is marked with a bold line. All models were controlled for covariates of age, sex, occupation, and diagnosis of major depressive disorder and bipolar disorder.

Figure 1 illustrates the results of our final model using multiple-mediator analysis. Dichotomized conduct problems exerted no direct effects on both suicide plans and suicide attempts (panel A), but were significantly associated with both extraversion and neuroticism. Neuroticism was significantly associated with both suicide plans and suicide attempts, whereas extraversion was not. Thus, neuroticism mediated the relationship between conduct problems and suicidality (indirect effect, suicide plan: OR = 1.30, BCA 95% CI = 1.04–1.83; suicide attempt: OR = 1.27, BCA 95% CI = 1.05–1.66), but extraversion showed no mediating effect (Table 4). Additionally, the continuous conduct problems (Panel B in Fig. 1) had a direct effect on the risk of suicide attempts (OR = 1.13, BCA 95% CI = 1.00–1.28). Overall, conduct problems at a young age exerted minimal direct effect on adulthood suicidality. However, conduct problems in youth increased the risk of adulthood suicidality through the indirect effects of higher neuroticism.

**Table 4:** Indirect effects of conduct problems on suicidality mediated by extraversion and neuroticism.

	Indirect effect			
	Suicide plans		Suicide attempts	
	OR	BCA 95% CI	OR	BCA 95% CI
Dichotomous (≥ 3)				
Total	1.24	(0.89–1.83)	1.27	(0.99–1.67)
Extraversion	0.96	(0.74–1.17)	1.00	(0.86–1.15)
Neuroticism	1.30	(1.04–1.83)	1.27	(1.05–1.66)
Continuous				
Total	1.03	(0.95–1.10)	1.03	(0.98–1.09)
Extraversion	0.98	(0.92–1.03)	0.99	(0.96–1.02)
Neuroticism	1.05	(1.01–1.12)	1.04	(0.99–1.09)

BCA 95% CI: Bias-corrected and accelerated 95% confidence intervals.

To evaluate the impact of the stringency of the definitions of conduct problems on the main findings, we performed a sensitivity analysis by applying a cutoff of 4 (Table S2). The results showed no intervening effects from extraversion on suicidality. However, neuroticism consistently intervened in the association between conduct problems and suicide plans (OR = 1.27, BCA 95% CI = 1.01–1.82).

## Discussion

Our study results support the hypothesis that adults who had conduct problems in youth had an increased risk of developing suicidal plans and attempts during adulthood compared with those without conduct problems. Having a diagnosis of mood disorders increased the risk of suicidality. However, the suicidality among those with conduct problems were not solely originated from the development of mood disorders. This finding was observed when we included the diagnosis in analytical models. The independent effect of conduct problems on suicidality was mainly mediated by the personality trait of neuroticism rather than by extraversion. Our study is the first to report the key role of personality traits in the relationship between CD and suicidality, as revealed by analysis including mood disorders as a covariate.

Our data support the findings of previous studies, which showed that both conduct problems and mood disorders are risk factors for suicidality^9,11,18,32^. However, the diagnosis of mood disorders was made in adulthood in our study and did not contribute to the influence of conduct problems on suicidality. A growing body of evidence suggests the association between adolescent depression and adult suicidality^33–38^, whereas other studies have not found such an association^8,39^. The stigmatization of suicide can lead to reporting bias and reduce the power to reveal the true associations^40^. In adolescents with conduct problems, the effects of depression on suicidal behaviors are less clear. Some studies have shown the independent effects of adolescents’ depressive symptoms and conduct problems on the risk of recurrent suicidal ideation or attempts^9^. A study in Taiwan that used the National Health Insurance Research Database reported that the risk of suicide attempts among adolescents with CD was high, even after adjustment for comorbid mood disorders^18^. Other studies have suggested interaction effects among suicide attempts, CD, and mood disorders. Goldston et al.^11^ found that CD only increased the risk of suicide attempts in the presence of depression. However, these studies have used pediatric mood disorder as a variable instead of confirmed diagnosis of mood disorders in adulthood. Recent cohort studies have shown that children diagnosed with depression might not be diagnosed with MDD in adulthood, and the most common disorder was minor depression instead^41–43^. Although the present study did not further explore the specific effect for individual mood disorders, the findings of the aforementioned studies indicate the complex pathologies of pediatric depression, adulthood depression, and emotional dysregulation in the development trajectory of adolescents^44^.

Our study revealed sex differences for the effects of conduct problems on suicidal plans and attempts. Men had more suicidal plans only, and women had more suicidal attempts. Because the majority of participants reported both suicidal plans and attempts, but not suicidal plans only, a high overall risk of developing suicidal behaviors (including both suicidal plans and attempts) was found in female participants in the current study. However, the sex influence was not consistent in previous studies. An early study reported that female participants with CD had an increased risk of both suicide attempts and mood disorder diagnosis despite the under-representativeness of female participants in their cohort^32^. By contrast, one study reported that male participants with CD were prone to suicide attempts and subsequently became suicide victims^45^, and the Finnish cohort study reported no predictability of conduct problems for suicidality in female participants^8^. The trajectories of suicidality from young through adulthood could be sex-specific and diverse according to a previous cohort study^16^. Considering the potential influence of different sexes, we adjusted for this variable in the final mediation model, and the mediating effect of neuroticism on suicidal behaviors still holds.

In Eysenck’s original proposition, children with conduct problems are more likely to demonstrate high extraversion and neuroticism^46^. According to Eysenck’s theory, individuals with high extraversion have lower levels of cortical arousal during aversive conditioning, are less sensitive to punishment, and have a higher tendency to exhibit antisocial behaviors. Furthermore, neuroticism strengthens the existing patterns of behavior; thus, individuals with high neuroticism often fail to conform to social norms once they develop conduct problems. Previous studies have shown findings inconsistent with this theory^26,47–49^, partly due to the small sample sizes and biased sex distribution. Although CD is believed to be an externalizing disorder^50,51^, our results revealed that CD increased both suicidal plans and attempts through neuroticism, a common internalizing factor that is usually strongly correlated with mood and anxiety disorders^51^. In contrast, the link was not mediated by extraversion, possibly because extraversion was found to be negatively related to hopelessness and indicated the tendency to experience positive emotions^52^. Children with CD might receive more attention if they exhibit more externalizing behaviors, reducing the risk of subsequent suicidality. Individuals exhibiting high internalizing behaviors are often overseen because they are less ‘visible’ and directly noticed through behavior to others as externalizing disorders. Early identification of their internalizing problems and even interventions targeting conduct problems and personality could further reduce the risk of suicidality among these adolescents as they grow into adulthood.

The present study has several strengths. The large sample size and representative data allowed us to perform mediation analysis. The longitudinal design and using well-validated instruments further enhanced the validity of this study. However, there were still some limitations. First, phenotypic data collection depends on self-reported CD symptoms and suicidality. Recall bias and stigmatization issues might prevent participants from fully disclosing their previous experiences. Second, data on conduct problems were collected retrospectively, and these problems were not diagnosed by psychiatrists in youth. Nevertheless, we performed analysis using both dichotomous diagnosis or continuous symptom numbers. We also performed sensitivity analysis, which showed similar and robust results with four conduct symptoms (Table S2). The conduct symptoms could not represent all conduct problems, which could compromise the validity of this study. Also, the CIDI module of suicidality we used could lead to information bias, which could be improved in future research by administering more accurate measures. By contrast, the diagnosis of mood disorders was made by psychiatrists thus enhance the validity of this study. However, other diagnoses including personality disorders were not included in our analysis. The accumulation of all mental illness might further interact with the personality traits and required future research. Third, previous twin studies have shown that the shared environment is an important component influencing suicide among adolescents with CD, but the socioeconomic data were limited in the original data collection. Finally, approximately two-thirds of the participants were diagnosed with mood disorders, and their suicidality was higher than that in the general population. Although we included healthy controls in our analysis, the generalizability of the data should be cautiously interpreted when applying to people without mood disorders. Also, the high mental health burden of CD and mood disorders per se could lead to adult adverse psychiatric outcomes^53^. The p-factor of psychopathology was not examined in our study, thus the impact of both internalizing and externalizing disorders could not be overlooked simply based on our results^54,55^.

In conclusion, compared with people who reported no conduct problems in youth, adults with conduct problems in youth exhibited a higher risk of suicidal plans and attempts. Mood disorders further increased suicidal risk, independent of conduct problems. The relationship between youth conduct problems and adulthood suicidality exerted minimal direct effect after personality traits were included in the model. The indirect effect of neuroticism might be a pivotal factor in the relationship between conduct problems and suicidal behaviors even after the diagnosis of mood disorders was controlled for. Further studies are warranted to clarify the complex relationship among conduct disorder, mood disorder, personality, and suicidality. Interventions targeting the personality construct might reduce the risk of suicide in people with conduct problems.

## Supplementary Information

Supplementary Information.

## Data Availability

The datasets used and/or analyzed during the current study available from the corresponding author on reasonable request.
