# Supplementary material for: The mediating effects of personality traits on the relationship of youth conduct problems and mood disorders with adulthood suicidality
Source: Sci Rep. 2023 Mar 15;13:4292. doi: 10.1038/s41598-023-31338-9 (PMC10017672; doi:10.1038/s41598-023-31338-9)
Supplement: Supplementary file 1 — Supplementary Information. [file 41598_2023_31338_MOESM1_ESM.docx]

Figure S1. The response to CIDI suicide modules


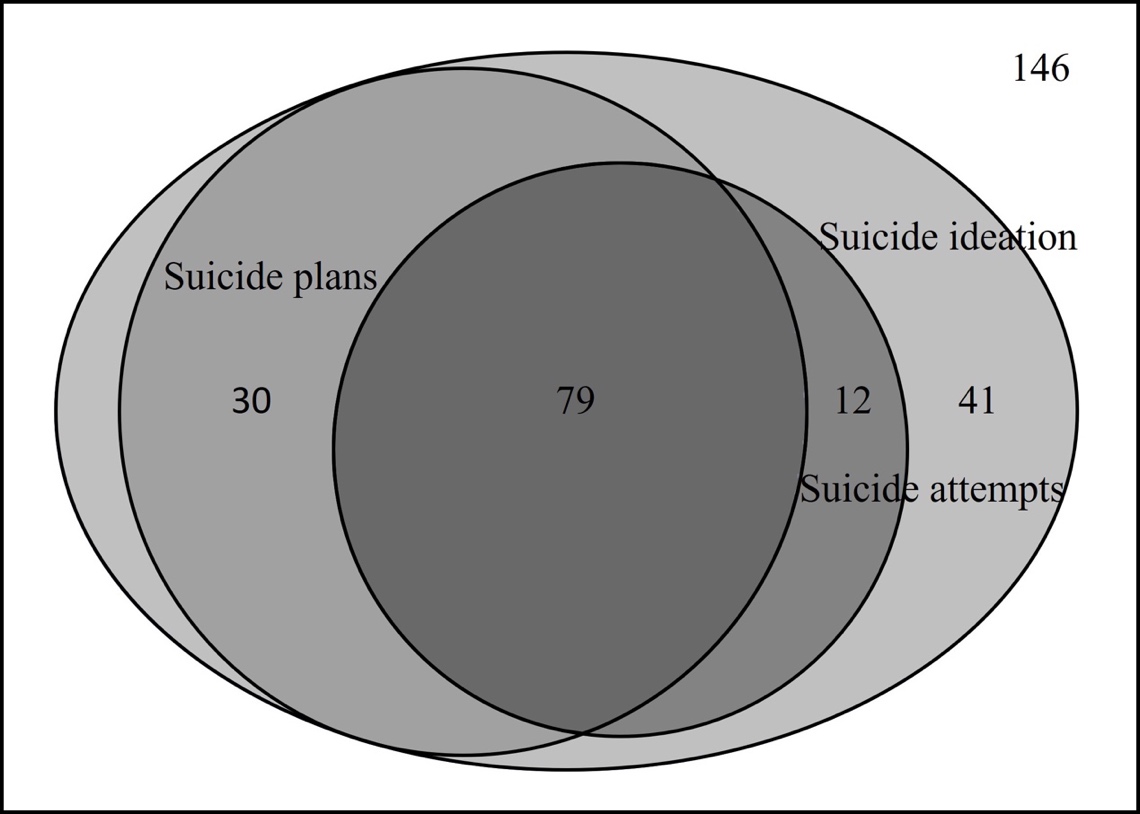


| Table S1. The frequencies of individual items of conduct problems by suicide experience | | | | | |
| --- | --- | --- | --- | --- | --- |
|  | | Suicide experience | | | |
| Item | Description | Total | No suicide experience | Suicide plans | Suicide attempts |
|  |  | (n=308) | (n=187) | (n=30) | (n=91) |
|  |  | (n, %) | (n, %) | (n, %) | (n, %) |
| **Aggression to People and Animals** | |  |  |  |  |
| 1. | Bullies, threatens, or intimidates others | 34 (11.0) | 14 (7.5) | 5 (16.7) | 15 (16.5) |
| 2. | Initiates physical fights | 49 (15.9) | 23 (12.3) | 7 (23.3) | 19 (20.9) |
| 3. | Used a weapon that can cause serious physical harm to others | 31 (10.1) | 10 (5.3) | 5 (16.7) | 16 (17.6) |
| 4. | Physically cruel to people | 26 (8.4) | 11 (5.9) | 6 (20.0) | 9 (9.9) |
| 5. | Physically cruel to animals | 32 (10.4) | 16 (8.6) | 4 (13.3) | 12 (13.2) |
| 6. | Stolen while confronting a victim | 19 (6.2) | 10 (5.3) | 3 (10.0) | 6 (6.6) |
| 7. | Forced someone into sexual activity. | 3 (1.0) | 0 (0.0) | 1 (3.3) | 2 (2.2) |
| **Destruction of Property** | |  |  |  |  |
| 8. | Deliberately engaged in fire setting | 10 (3.2) | 2 (1.1) | 2 (6.7) | 6 (6.6) |
| 9. | Deliberately destroyed others’ property | 43 (14.0) | 22 (11.8) | 6 (20.0) | 15 (16.5) |
| **Deceitfulness or Theft** | |  |  |  |  |
| 10. | Broken into someone else’s house, building, or car. | 27 (8.8) | 14 (7.5) | 3 (10.0) | 10 (11.0) |
| 11. | Lies to obtain goods or favors or to avoid obligations | 146 (47.4) | 85 (45.5) | 14 (46.7) | 47 (51.6) |
| 12. | Stolen items of nontrivial value without confronting a victim | 126 (40.9) | 77 (41.2) | 15 (50.0) | 34 (37.4) |
| **Serious Violations of Rules** | |  |  |  |  |
| 13. | Stays out at night despite parental prohibitions | 110 (35.7) | 58 (31.0) | 11 (36.7) | 41 (45.1) |
| 14. | Run away from home overnight | 55 (17.9) | 20 (10.7) | 6 (20.0) | 29 (31.9) |
| 15. | Truant from school | 109 (35.4) | 59 (31.6) | 7 (23.3) | 43 (47.3) |
|  |  |  |  |  |  |
| **Cumulative items ≥ 3** | | 133 (43.2) | 76 (40.6) | 13 (43.3) | 44 (48.4) |

| Table S2. Indirect effects of conduct problems on suicidality mediated by extraversion and neuroticism | | | | | |
| --- | --- | --- | --- | --- | --- |
|  | Indirect effect | | | | |
|  | Conduct problems ≥ 4 | | | | |
|  | Suicide plans | |  | Suicide attempts | |
|  | OR | BCA 95% CI |  | OR | BCA 95% CI |
| Total | 1.19 | (0.85-1.80) |  | 1.21 | (0.93-1.60) |
| Extraversion | 0.93 | (0.73-1.16) |  | 1.00 | (0.87-1.16) |
| Neuroticism | 1.27 | (1.01-1.82) |  | 1.20 | (0.98-1.57) |
| BCA 95% CI: Bias-corrected and accelerated 95% confidence intervals | | | | | |
